# Interactions between *Trypanosoma cruzi* Secreted Proteins and Host Cell Signaling Pathways

**DOI:** 10.3389/fmicb.2016.00388

**Published:** 2016-03-31

**Authors:** Renata Watanabe Costa, Jose F. da Silveira, Diana Bahia

**Affiliations:** ^1^Departamento de Microbiologia, Imunologia e Parasitologia, Escola Paulista de Medicina, Universidade Federal de São PauloSão Paulo, Brazil; ^2^Departamento de Biologia Geral, Instituto de Ciências Biológicas, Universidade Federal de Minas GeraisMinas Gerais, Brazil

**Keywords:** *T. cruzi*, secretome, secreted proteins, virulence factor, host parasite interaction, host cell signaling, host cell invasion, Chagas disease

## Abstract

Chagas disease is one of the prevalent neglected tropical diseases, affecting at least 6–7 million individuals in Latin America. It is caused by the protozoan parasite *Trypanosoma cruzi*, which is transmitted to vertebrate hosts by blood-sucking insects. After infection, the parasite invades and multiplies in the myocardium, leading to acute myocarditis that kills around 5% of untreated individuals. *T. cruzi* secretes proteins that manipulate multiple host cell signaling pathways to promote host cell invasion. The primary secreted lysosomal peptidase in *T. cruzi* is cruzipain, which has been shown to modulate the host immune response. Cruzipain hinders macrophage activation during the early stages of infection by interrupting the NF-kB P65 mediated signaling pathway. This allows the parasite to survive and replicate, and may contribute to the spread of infection in acute Chagas disease. Another secreted protein P21, which is expressed in all of the developmental stages of *T. cruzi*, has been shown to modulate host phagocytosis signaling pathways. The parasite also secretes soluble factors that exert effects on host extracellular matrix, such as proteolytic degradation of collagens. Finally, secreted phospholipase A from *T. cruzi* contributes to lipid modifications on host cells and concomitantly activates the PKC signaling pathway. Here, we present a brief review of the interaction between secreted proteins from *T. cruzi* and the host cells, emphasizing the manipulation of host signaling pathways during invasion.

## Introduction

### *Trypanosoma cruzi*: Life Cycle and Chagas Disease

Chagas disease is caused by the protozoan parasite *Trypanosoma cruzi*, affects 6–7 million individuals, primarily in Latin America, and is associated with negative economic impacts in developing countries (http://www.who.int/mediacentre/factsheets/fs340/en/). *T. cruzi is* transmitted to vertebrate hosts by the triatomine vector *Triatoma infestans*. Despite its high incidence and economic costs, Chagas disease remains a neglected tropical disease; it does not have an effective pharmacological treatment and there are minimal investments in finding a cure for Chagas disease (Clayton, 2010; Souza et al., 2010). The life cycle of *T. cruzi* has four developmental phases that occur in the hematophagous insect vector and bloodstream and tissues of mammalian hosts (Souza et al., 2010). The epimastigote (EPI) is a non-infectious replicative form found in the vector’s digestive tract. The EPI differentiates into the metacyclic trypomastigote (MT), which is transmitted to mammals through the insect’s feces during a blood meal or by the oral route. The MT invade mammalian host cells where they transform into an amastigote (AMA) that replicates intracellularly. After a multiple rounds of replication, the AMAs differentiate back into trypomastigotes (TCTs), which are released into the extracellular milieu when the host cell is disrupted. TCTs can invade neighboring host cells or be released into the blood stream where they can infect other tissues or be ingested by a feeding insect. Once the host has been infected, the parasite can invade and multiply in the myocardium, leading to acute myocarditis, which kills around 5% of untreated individuals (Ponce et al., 2013).

Similar to other intracellular protozoa, *T. cruzi* is an intracellular parasite that invades different types of cells to evade the host immune system (Guiñazú et al., 2007). Intracellular parasites have complex lifecycles that involve several developmental stages, and usually contain multiple secreted proteins that can manipulate host cell signaling pathways to promote parasite adhesion, recognition, and invasion (Burleigh and Woolsey, 2002). The complex interplay between proteins secreted by *T. cruzi* that affect the host cell environment or contribute to immune evasion likely influences the outcome of infection. Understanding the role of secreted proteins during *T. cruzi* infection is critical to deepen the knowledge of the pathogenesis of Chagas disease (McConville et al., 2002).

### *T. cruzi* Secretome

In eukaryotes, secreted proteins typically contain an N-terminal signal peptide that directs them to the classical endoplasmic reticulum (ER)/Golgi-dependent secretion pathway. Secretory proteins that do not contain the signal peptide are secreted outside the plasma membrane using non-classical secretory pathways including, membrane-bound extracellular vesicles (EVs), such as exosomes and ectosomes (Nickel and Seedorf, 2008; Simpson and Mathivanan, 2012). Only a small fraction (∼9%) of the proteins in the *T. cruzi* secretome contain an N-terminal signal peptide suggesting that they are secreted by classical pathways (Bayer-Santos et al., 2013), the remaining proteins are likely secreted by non-classical pathways (Torrecilhas et al., 2009, 2012; Bayer-Santos et al., 2013; Marcilla et al., 2014).

Secretion or shedding of EVs by *T. cruzi* can occur spontaneously or be induced by nutritional or chemical stress (da Silveira et al., 1979; Torrecilhas et al., 2009, 2012; Bayer-Santos et al., 2013; Marcilla et al., 2014). A considerable number of the *T. cruzi* secreted/excreted proteins have been characterized at the structural and functional levels. Some of the secreted *T. cruzi* proteins, such as the *trans*-sialidase (TS) glycoproteins (TS/SAPA, Tc85, gp82, gp90, CRP, TESA), mucin-associated surface proteins (MASP), cruzipain, gp63, mucins, and serine-, alanine-, and proline-rich proteins (SAP), are associated with the plasma membrane via a glycosylphosphatidylinositol (GPI) anchor (Torrecilhas et al., 2009, 2012; Bayer-Santos et al., 2013; Marcilla et al., 2014). Several of these proteins (e.g., TS/SAPA, CRP, mucins) are also spontaneously shed from the parasite surface in a soluble form that lacks the GPI anchor, possibly due to cleavage by an endogenous phospholipase C (Affranchino et al., 1989; de Almeida and Heise, 1993; Bartholomeu et al., 2009; de Pablos et al., 2011; Cánepa et al., 2012a). Others (e.g., Tc85) are shed with the GPI anchor linked to membrane vesicles (Zingales et al., 1985; Abuin et al., 1996a).

Trypomastigotes and AMAs release EVs containing virulence factors involved in: (i) host cell invasion and intracellular parasite development, such as the TS and TS-like proteins (Zingales et al., 1985; Gonçalves et al., 1991; Schenkman et al., 1991; Abuin et al., 1996a,b; Torrecilhas et al., 2009, 2012; Cánepa et al., 2012a; Maeda et al., 2012; Bayer-Santos et al., 2013; Marcilla et al., 2014; Mattos et al., 2014), peptidyl prolyl *cis-trans*-isomerase (Moro et al., 1995), oligopeptidases and proteases (Meirelles et al., 1992; Caler et al., 1998; Scharfstein et al., 2000; Cuevas et al., 2003; Bastos et al., 2005; Doyle et al., 2011; Maeda et al., 2014), phospolipases A1 and C (Andrews et al., 1988; Rosenberg et al., 1991; Furuya et al., 2000; Okura et al., 2005; Belaunzarán et al., 2007, 2013; Castillo et al., 2013); mucins and mucin-like proteins (Gazzinelli et al., 1991; de Diego et al., 1997; Kierszenbaum et al., 1999; Pereira-Chioccola et al., 2000; Kierszenbaum et al., 2002; Alcaide and Fresno, 2004; Cánepa et al., 2012b), MASP (de Pablos et al., 2011; Cánepa et al., 2012a), SAP (Baida et al., 2006; Zanforlin et al., 2013), P21 AMA specific proteins (da Silva et al., 2009), surface membrane proteins (TcSMP; Martins et al., 2015); (ii) immune evasion (Andrews et al., 1990; Norris et al., 1991; Norris and Schrimpf, 1994; Ouaissi et al., 1995; Reina-San-Martin et al., 2000; Martin et al., 2006; Mott et al., 2011; Kulkarni et al., 2013; Nogueira et al., 2015); and (iii) increased heart parasitism, inflammation, and arrhythmia that contribute to the pathogenesis of Chagas disease (Torrecilhas et al., 2009; Nogueira et al., 2015; Rodriguez-Angulo et al., 2015). In addition, some of the secreted/excreted proteins are diagnostic markers for Chagas disease (Jazin et al., 1995; Umezawa et al., 1996; Agusti et al., 2000; Bernabó et al., 2013). This mini review will focus on specific molecules secreted by *T. cruzi* that have already been identified as interfering with host cell signaling and that ultimately play a role in the ability of *T. cruzi* to evade the immune system.

### *T. cruzi* Cruzipain: A Role in Evading the Host Immune Response and Promoting Survival in Cardiomyocytes

To facilitate their entry into non-phagocytic cells, infectious TCTs employ an arsenal of surface glycoproteins, secreted proteases, and signaling agonists to actively manipulate multiple host cell signaling pathways (Burleigh and Woolsey, 2002). Several studies using synthetic irreversible cysteine peptidase inhibitors have demonstrated that *T. cruzi* infectivity, host immune evasion, and intracellular growth depend on the activity of cruzipain (Meirelles et al., 1992; Waghabi et al., 2005; McKerrow et al., 2008). To facilitate entry into non-phagocytic cells like endothelial cells and cardiomyocytes, cruzipain acts on a cell-bound kininogen to generate bradykinin, which upon recognition by the B2 bradykinin receptor, triggers the Ca^2+^ mobilization required for parasite internalization (Scharfstein et al., 2000; Guiñazú et al., 2007; Maeda et al., 2014).

Murine macrophages stimulated with cruzipain up-regulate arginase activity and increase production of IL-10 and TGF-β, thereby increasing *T. cruzi* survival (Stempin et al., 2002). TGF-β in particular can suppress some of the microbicidal functions of macrophages and is one way that parasites create a favorable cellular microenvironment to gain a survival advantage (Gantt et al., 2003; Waghabi et al., 2005). Previous studies have demonstrated that forms of *T. cruzi* are able to activate latent TGF-β (Waghabi et al., 2005). Treatment of macrophages with increasing doses of cruzipain promoted the activation of TGF-β in a dose-dependent manner, confirming that this peptidase is capable of activating latent TGF-β in the absence of any other host or parasite factors (Ferrão et al., 2015). In addition, transgenic EPIs overexpressing chagasin, a natural cruzipain inhibitor, were significantly less able to activate latent TGF-β when compared to wild type parasites (Santos et al., 2005; Ferrão et al., 2015).

The role of cruzipain in cell entry and TGF-β production suggest that it may function during the early events of macrophage infection to facilitate parasite survival and replication. Taken together, the data suggests that cruzipain is a potential pharmaceutical target as it may have an essential role in the pathogenesis of Chagas disease (Guiñazú et al., 2007; Doyle et al., 2011). Based on this evidence, cruzipain inhibitors are considered promising anti-*T. cruzi* chemotherapeutic agents (Ndao et al., 2014; Branquinha et al., 2015). Irreversible cruzipain inhibitors, such as the prototype molecule K777 (also known as K11777) have been efficacious in experimental models of *T. cruzi* infection (Engel et al., 1998; Barr et al., 2005; Doyle et al., 2011).

In parallel to the immunological findings, cruzipain promotes cardiomyocyte survival via the PI3K and MEK1-dependent signaling pathways (Aoki et al., 2004, 2006). Cardiomyocytes were pretreated with PI3K or MAPK inhibitors and grown in the presence or absence of cruzipain. Cardiomyocyte apoptosis was decreased after cruzipain treatment, but this protective effect was reduced by incubation with PI3K and MEK1 inhibitors, which had no effect on cruzipain-mediated cardiomyocyte survival in the absence of cruzipain. These findings suggest the survival effects of cruzipain are regulated by effector proteins downstream of PI3K and MEK1. Moreover, *T. cruzi* infection as well as cruzipain itself mediates the phosphorylation of ERK1/2 and Akt, and cruzipain inhibits proteolytic cleavage of caspase 3 via PI3K and MEK1-dependent signaling pathways (Fujio et al., 2000; Aoki et al., 2006). Together the data strongly suggest cruzipain mediates survival in part via anti-apoptotic PI3K/MEK1 signaling. Another study has shown that the anti-apoptotic effect of cruzipain is also mediated in part by arginase activity and Bcl-2 expression (Aoki et al., 2004). Thus, cruzipain activates at least two signaling pathways leading to enhanced cardiomyocyte survival. Parallel activation of these signal transduction pathways may represent a cellular strategy to amplify survival signals in the target cell. Elucidating the pro-survival pathways may lead to a better understanding of the parasite–host relationship and may provide useful targets for the treatment of Chagas disease (Aoki et al., 2004, 2006).

### *T. cruzi* Phospholipase A1: A Role in Activating Host Protein Kinase C (PKC) Throughout Infection

Phospholipases play a critical role in some physiological processes including the generation of signaling lipids that are relevant to disease (Dennis, 2015). In the case of *T. cruzi*, phospholipid degrading enzymes are associated with the inflammatory responses elicited by degenerating AMA nests in the tissues of patients with Chagas disease (Wainszelbaum et al., 2001).

**FIGURE 1:**
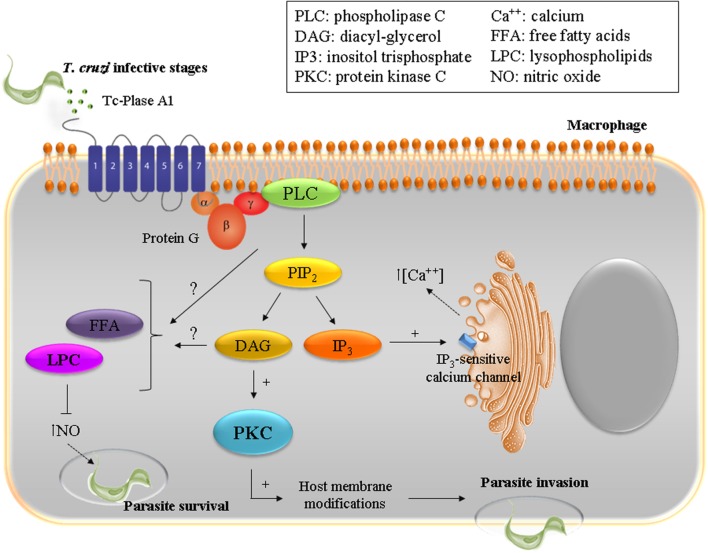
**To ensure successful invasion of the host cell *T. cruzi* has developed a multi-step process with redundant mechanisms involving diverse host and parasite molecules.** The enzyme Plase A1 (Tc-Plase A1) is secreted by *T. cruzi* and is widely present during the infectious life stages. Tc-Plase A1 may contribute to lipid modifications on host cells and concomitantly activates the PKC signaling pathway. This suggests that Tc-Plase A1 is involved in the early events of the parasite–host cell interaction and precedes parasite invasion.

Throughout its life cycle, *T. cruzi* has to adapt to different environments through morphological and functional changes that involve complex networks of enzymatic pathways, including phospholipases. *T. cruzi* Phospholipase A1 (Tc-PLA1) is secreted by the parasite into the extracellular medium and shows remarkably higher membrane-bound activity in infectious AMAs and TCTs (Wainszelbaum et al., 2001; Belaunzarán et al., 2007). In VERO cells, treatment with Tc-PLA1 and PMA (phorbol 12-myristate 13-acetate), a known PKC activator, demonstrated that Tc-PLA1 is involved in host cell lipid modifications leading to PKC activation (34). Tc-PLA1 significantly modified the host cell lipid profile by generating secondary lipid messengers (DG, FFA, and LPC) and concomitant PKC activation. PKC has been implicated in increased parasite invasion, suggesting that Tc-PLA1 is involved in the early events of parasite–host cell interaction preceding parasite invasion (Belaunzarán et al., 2007). Specific anti- Tc-PLA1 antibodies can bind to the surface of the parasite and neutralize Tc-PLA1 activity, preventing parasite invasion. This suggests that Tc-PLA1 is an emerging virulence factor for *T. cruzi* and emphasizes the promise of Tc-PLA1 as a potential therapeutic target (Belaunzarán et al., 2007). Taking these findings into consideration, Tc-PLA1-mediated host cell PKC activation could modulate Ca^2+^ release from intracellular stores thereby contributing to parasite invasion. Ca^2+^ mobilization, in both host cell and parasite, is required during the internalization process (Villalta et al., 1999; Yoshida, 2006; Souza et al., 2010; Maeda et al., 2012). In addition, *T. cruzi* infective stages partially incorporated and metabolized LPC, therefore the remaining extracellular LPC might exert a toxic effect on the host cell, reinforcing the involvement of Tc-PLA1 in the pathogenesis. In this concern, it has been described that LPC inhibits nitric oxide production by *T. cruzi* stimulated macrophages (**Figure 1**), and thus interferes with the vertebrate host immune system (Belaunzarán et al., 2007).

### Secreted *T. cruzi* Cyclophilin Inactivates the Lytic Vector Defense

*Trypanosoma cruzi* not only has to interact with the mammalian host but also with its insect vector (*Triatoma infestans*), and many of these interactions are still unknown. Innate immune cationic antimicrobial peptides (CAMPs) are expressed by a wide variety of insects to prevent microbial colonization and infection. Several CAMPs have been identified from the saliva, hemolymph, and intestinal tract of reduviid insects (Lopez et al., 2003). Kulkarni et al. (2013) studied the interactions between CAMPs and *T. cruzi*, and found a unique parasite-driven pathway that modified host CAMPs. Parasites exposed to cyclophilin-trialysin have enhanced binding and invasion in myoblasts pre-grown leading to higher infectivity. They found that secreted parasite cyclophilin, a peptidyl-prolyl isomerase involved in protein folding (Kulkarni et al., 2013; Carraro et al., 2015), binds to and inactivates trialysin via its proline residue. Replicating insect-stage parasites secrete cyclophilin 19 as they migrate through the reduviid gastrointestinal tract. Cyclophilin 19 binds to and isomerizes the CAMP peptide neutralizing its anti-parasitic activity. The cyclophilin-trialysin complex then synergistically acts on the parasites to activate calcineurin phosphatase signaling, which drives metabolic activation and ATP production leading to enhanced infectivity. This parasite pathway is a mechanism of CAMP recognition, evasion, and adaptation mediated through calcineurin intracellular signaling (Kulkarni et al., 2013; Carraro et al., 2015). These findings also represent one of the few descriptions of specific stimuli that enhance infectivity of *T. cruzi* and indicate a defined host molecule-based environmental sensing mechanism in this group of organisms (Kulkarni et al., 2013).

### *T. cruzi* Soluble Factors: Effects on the Host Extracellular Matrix

Trypomastigotes trigger rapid changes in the host cell signaling pathways during their early interactions with mammalian host cells to facilitate the process of parasite entry into non-professional phagocytic cells (Giddings et al., 2006; Yoshida, 2008). However, *T. cruzi* also affects the host cell downstream of the invasion process. Transcriptional profiling of *T. cruzi*-infected fibroblasts showed that the earliest detectable changes triggered by infectious TCTs involved downregulation of a small subset of genes including members of the CCN family (cyr61 and ctgf/ccn2) that play critical roles in cardiovascular development (angiogenesis), injury repair, fibrotic disease, and extracellular matrix (ECM) homeostasis (Chen and Lau, 2009; Mott et al., 2011). Connective tissue growth factor (CTGF/CCN2) promotes cell proliferation and cooperates with TGF-ß to promote myofibroblast differentiation and enhanced ECM synthesis. Mott et al. (2011) showed that *T. cruzi* may release a factor that inhibits TGF-ß-mediated expression of CTGF/CCN2. The expression of CTGF/CCN2 is also controlled by the ETS family of transcriptional factors, which are regulated through MAP kinase signaling. *T. cruzi*-dependent abrogation of CTGF/CCN2 expression in human dermal fibroblasts is associated with inhibition of both basal and agonist-induced activation of MAP kinase signaling (Mott et al., 2011). *T. cruzi*-mediated down-regulation of CTGF expression requires *de novo* host cell protein synthesis, indicating that the ability of *T. cruzi* to interfere with the host fibrogenic response is a complex process requiring input from multiple host cell signaling pathways (Unnikrishnan and Burleigh, 2004; Mott et al., 2011).

Regarding the impact of *T. cruzi* secreted factors on TGF-ß-induced fibroblast gene expression, a discrete subset of agonist-inducible fibroblast genes are sensitive to factors secreted/released by *T. cruzi*. A study reports that the group of TGFß-inducible genes that exhibit the highest sensitivity to a *T. cruzi* secreted/released fraction are MAP kinase-regulated genes that function in wound repair, ECM remodeling, and host response pathways. Inhibition of ECM synthesis because of these secreted parasite factors would facilitate dissemination from early sites of infection (Mott et al., 2011).

### Secreted *T. cruzi* P21 Enhances Host Phagocytosis

P21 is a secreted protein expressed in all of the developmental stages in the *T. cruzi* lifecycle that may play an important role in parasite internalization (da Silva et al., 2009). Rodrigues et al. (2012) engineered a recombinant protein based on P21 (P21-His6) and then assessed its ability to upregulate phagocytosis in macrophages and alter host cell signaling. P21-His6 upregulated phagocytosis in macrophages in a manner dependent on CXCR4-binding and actin polymerization, and triggered the PI3K signaling pathway (Rodrigues et al., 2012). P21-His6 required PI3K signaling independent of AKT for its function (Vasudevan et al., 2009; Lee et al., 2011). PI3K-dependent signal transduction through the Rho-family GTPases occurs during FcR-mediated phagocytosis and that PI3K-dependent deactivation of Cdc42 is necessary for phagocytosis. Moreover, the activities of PI3K and Cdc42 are linked: FcR-activated Cdc42 stimulates PI3K, which increases concentrations of PI(3,4,5)P3 in phagocytic cups, allowing the PI(3,4,5)P3-dependent deactivation of Cdc42 that is necessary to complete phagocytosis (Beemiller et al., 2010). In addition, previous work has provided evidence of PI3K activation in non-professional phagocytic cells during *T. cruzi* cell invasion (Woolsey et al., 2003; Rodrigues et al., 2012). In sum, P21 serves as part of the host cell invasion machinery by triggering actin polymerization on the host cell through interactions with the CXCR4 chemokine receptor on the cell membrane, and favoring its own phagocytosis into the host (dos Santos et al., 2014).

### *T. cruzi* MASP: A Role in Evading Host Immune Cells

The annotation of the *T. cruzi* genome revealed a new multigene family composed of approximately 1,300 genes, which became known as MASPs because they were clustered with genes encoding mucins and other surface protein families (El-Sayed et al., 2005). MASP proteins are GPI-anchored glycoproteins expressed on the surface of the circulating infectious forms of the parasite that can be secreted into the extracellular medium (Bartholomeu et al., 2009; dos Santos et al., 2012; Serna et al., 2014). MASP is the second largest gene family (1377 genes and 433 pseudogenes), representing approximately 6% of the T. cruzi genome (Serna et al., 2014).

dos Santos et al. (2012) using antibody recognition of several MASP peptides observed the interaction of these proteins with the host immune system during acute *T. cruzi* infection. The MASP family may play a role in promoting the polyclonal lymphocyte activation that leads to hypergammaglobulinemia and the delayed specific humoral immune response, characteristic of the acute phase of Chagas disease. Polyclonal B-cell activation might diffuse the immune response, preventing the development of a specific and neutralizing response against the parasite and its complete elimination. Additionally, MASP peptides could possibly mediate both specific T-cell dependent and non-specific T-cell independent immune responses. This hypothesis is partially supported by the differential recognition of MASPs by immunoglobulin (Ig) M and IgG and the difference in the antibody affinity levels against each of the synthetic peptides. All of these phenomena are suggestive of an immune evasion mechanism (Reina-San-Martin et al., 2000; Minoprio, 2001; Gao et al., 2002; dos Santos et al., 2012).

## Concluding Remarks

Host cell invasion and parasite internalization are important steps in the evolution of parasitism by several pathogens. These processes present at least two important advantages: protection against the host immune response and access to a microenvironment rich in metabolic products (Barrias et al., 2012). Substantial progress has been made in understanding the roles of secreted proteins in infection and invasion by pathogenic *T. cruzi*. Host cell intracellular signaling can combat the infection; but it can also favor parasite entry. Parasites hijack the host immune response, phagocytosis, ECM, and anti-parasitic proteins for their own survival, replication, and immune evasion purposes. The complex networks are interconnected and require extensive study to identify intracellular rearrangements that facilitate parasite internalization; the tools in use today include bioinformatics, novel molecular level studies, and new experimental drugs. A multidisciplinary approach to understanding parasite host interaction will be critical to better understand *T. cruzi* physiopathology, diagnosis, and treatment.

## Author Contributions

All authors listed conceived and wrote the manuscript.

## Conflict of Interest Statement

The authors declare that the research was conducted in the absence of any commercial or financial relationships that could be construed as a potential conflict of interest.
